# Biparental incubation behaviour under temperature extremes in sandbank nesting black skimmers

**DOI:** 10.1002/ece3.11021

**Published:** 2024-02-15

**Authors:** Martin Austad, Jørgen Sand Sæbø, Ronny Steen, Katharine S. Goodenough, Lisa Davenport, Torbjørn Haugaasen

**Affiliations:** ^1^ Faculty of Environmental Sciences and Natural Resource Management Norwegian University of Life Sciences Ås Norway; ^2^ Department of Animal Ecology & Systematics Justus‐Liebig University Giessen Giessen Germany; ^3^ Biology Department University of Oklahoma Norman Oklahoma USA; ^4^ Department of Biology and Florida Museum of Natural History University of Florida Gainesville Florida USA; ^5^ School of Science and Engineering James Cook University Cairns Queensland Australia

**Keywords:** Amazonia, *Rynchops niger*, thermal ecology, time‐lapse

## Abstract

Birds nesting on riverine beaches are exposed to large temperature fluctuations, while changing water levels pose flooding risks. We used miniature temperature loggers (*i*Buttons®) placed in nests and on the beach surface combined with time‐lapse photography to study incubation behaviour in the black skimmer (*Rynchops niger*) on the Manu River, Peru. Since the species exhibits sexual size dimorphism, we could identify partner switches in images and the contribution to incubation effort by each pair member. Results of the study documented that nest temperature was less affected by ambient temperature and fluctuated less than the surroundings. Despite shorter incubation bouts at midday, black skimmers maintained a close to constant presence at the nest by more frequent nest exchanges. In fact, while female black skimmers generally incubated more and for longer than males, pairs shared incubation most consistently during the hottest part of the day. Incubation probability decreased around dusk, a peak foraging time for the species and a time when beach temperature overlapped with nest temperature. A biparental incubation strategy across the diel cycle appears to allow black skimmers breeding at the Manu River to incubate in challenging thermal conditions, but further studies are needed to determine proximity to thermal limits.

## Graphical abstract

We investigated biparental incubation behaviour and nest temperature using autonomous recording devices and test whether male and female time budgets change across the diel cycle in a sexually dimorphic species. Black skimmers made use of several behavioural mechanisms, such as shorter incubation bouts, higher pair synchronisation and thermoregulatory postures to maintain high nest attendance even in the hottest part of the day. Our findings can inform habitat management and conservation which are increasingly needed for sandbank nesting species.

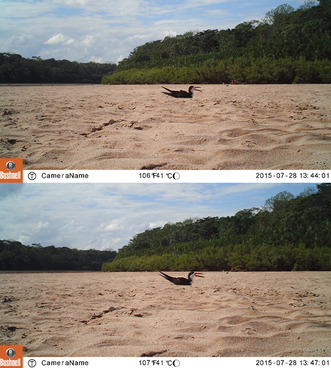

## 1 INTRODUCTION

Diel activity patterns in some animals are an adaptation to environmental variability across the day and night, including abiotic factors such as temperature (Kronfeld‐Schor et al., 2013). Due to the potential lethal or sub‐lethal effects of temperature, animals mediate their behaviour and habitat selection throughout the diel cycle in response to temperature fluctuations (Alonso et al., 2016; Maloney et al., 2005). Oviparous organisms must additionally ensure eggs are at suitable temperatures, which apart from the initial nest site selection in a favourable microclimate, can be mediated by parent behaviour (Carroll et al., 2018). Temperature regulation of eggs and nests by adults is especially important in birds, which generally lay eggs above ground resulting in higher potential exposure to environmental fluctuations (Carroll et al., 2015). Therefore, incubation is an essential part of avian reproduction by which adults actively maintain eggs in the narrow tolerable range of temperatures to ensure embryonic development and successful hatching (DuRant et al., 2013).

Several bird species nest on open unvegetated ground where eggs are laid directly on a substrate without insulating material. Moreover, vegetation or other structures that would offer shade are often avoided as an anti‐predation mechanism (Amat & Masero, 2004; Grant, 1982). This type of nest preparation and site selection often requires effort to maintain eggs cooler than surrounding temperatures during the day (Deeming, 2002). In such cases, adult birds do not necessarily carry out contact incubation, but have been shown to alternate between sitting less tightly on the eggs or even standing over the eggs (AlRashidi, 2016; Clauser & McRae, 2017; Grant, 1982). Exposed nests are at risk of failure from high solar radiation and ambient temperature, with rapid increases in egg temperature having been measured on adults leaving the nest during the day (Grant, 1982; Mougeot et al., 2014). However, nest attendance exposes adults to thermal stress (AlRashidi et al., 2010; DuRant et al., 2013). While changes in posture can promote mechanisms of evaporative cooling by exposing the head and beak to convection and together with panting and gular fluttering can prolong incubation bouts (Bartholomew & Dawson, 1979; Cook et al., 2020; Grant, 1982; Walsberg & Voss‐Roberts, 1983), ultimately ground nesting birds might have to trade off nest attendance with cooling mechanisms carried out away from the nest (Amat & Masero, 2004).

One strategy in several ground nesting species is biparental incubation, by which one of the parents can relieve the other at the nest and decrease the conflict between attending and temporarily deserting the nest (AlRashidi et al., 2010; Bulla et al., 2015; DuRant et al., 2013). At the scale of species' range, biparental care does not appear related to environmental conditions but rather to adult sex ratios, requiring studies at finer scale to determine the role of ambient temperature (Remeš et al., 2015). Indeed, within biparental systems, the frequency of partners relieving each other at the nest, and length of incubation bouts, varies across the time of day with factors such as foraging efficiency and weather (Braimoh‐Azaki et al., 2023; Bulla et al., 2015).

The black skimmer (*Rynchops niger*) is a colonial, piscivorous bird species breeding on the American continent. Black skimmers are mostly coastal in the northern hemisphere, while in Amazonia it is one of the few species nesting on the unvegetated river beaches exposed during the dry season (Gochfeld, 1978; Terborgh, 1985; Vieira et al., 2018a). In these equatorial riverine environments, the species has to cope with high temperatures, high predation threat and flood risk (Davenport et al., 2016). Despite the flood hazard, black skimmers often nest close to the river edge in open scrapes in the sand (Zarza et al., 2013). A clutch is typically two to four eggs and incubated by both male and female for 20 to 25 days, starting as soon as the first egg is laid (Burger, 1981a; Dinsmore, 2008; Schuchmann et al., 2018; Vieira et al., 2018b).

Compared to other closely related species, the black skimmer exhibits pronounced sexual size dimorphism, with males averaging about 30% larger than females in measures of mass and exposed culmen, also distinguishable in images by visual observers (Vieira et al., 2018a). Clear size dimorphism allows for determining the contribution to incubation by each member of the pair, which is not always possible in other study species lacking evident sexual dimorphism (AlRashidi, 2016; AlRashidi & Shobrak, 2015). Incubation behaviour, especially in the Amazon breeding range of black skimmers, is poorly studied (Vieira et al., 2018b). Generally, females were found to incubate more than males (Quinn, 1990; Schuchmann et al., 2022), but one study from a North American population found higher male incubation rates (Burger, 1981b). However, the limited observation time in these studies, mainly constrained to daylight hours, means we know very little about the incubation behaviour across the entire diel cycle, especially nocturnal time allocation to incubation (Vieira et al., 2018b), which might conflict with foraging requirements (Bulla et al., 2015; Erwin, 1977). Closing the knowledge gap of how open beach nesting birds change incubation behaviour across the diel cycle is important in the face of climate change, which may challenge the strategies that birds use in temperature extremes (Clauser & McRae, 2017; Cook et al., 2020; Oswald & Arnold, 2012).

This study investigated black skimmer breeding behaviour and thermal ecology along the Manu River in the Peruvian Amazon. Specifically, with autonomous recording devices, we monitored incubation behaviours and nest temperatures throughout a full breeding season to determine diel patterns in behaviour and time budgets allocated by males and females. We expected that black skimmers maintain continuous nest attentiveness throughout the diel cycle and that nest temperatures are buffered irrespective of temperature fluctuations on the beach surface. Accordingly, we expected incubating black skimmers to show behavioural responses to any diel periods that might be thermally stressful, namely an increase in adoption of a stretched neck posture that promotes evaporative cooling at the nest (Bartholomew & Dawson, 1979). However, since such mechanisms might not be sufficient, we also expected shorter incubation bouts, which would allow for cooling away from the nest (Amat & Masero, 2004). Finally, for shorter incubation bouts not to affect continuous nest attentiveness, we predicted the contribution by male and female black skimmers to be most equal and synchronised during any thermally stressful diel periods.

## 2 METHODOLOGY

### 2.1 Study area

This study was carried out along 38 km of the Manu River, within the research zone of the Cocha Cashu Biological Station (11°53′17.38″ S, 71°24′27.02″ W, 350 masl) during the months of June to September 2015.

The close proximity of the study area to the Andes causes a more unpredictable flooding regime than in the central Amazon (Robinson & Terborgh, 1997), with flash floods regularly occurring even during the low‐water season due to heavy rainfall in the mountains. During the period of this study, two nesting attempts by black skimmers were disrupted by floods (9th July and 10th August). These floods inundated all the beaches in the study area and caused nest failure. Black skimmers made new nesting attempts as soon as floods receded.

**FIGURE A1:**
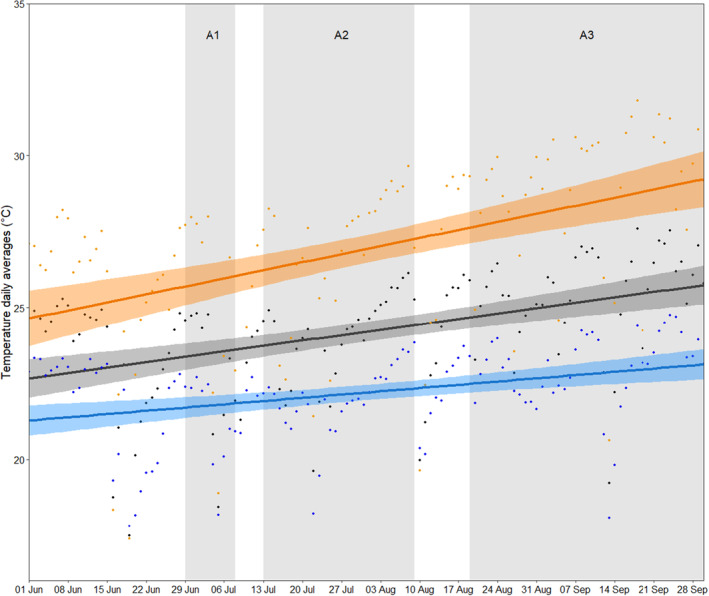
Mean daily temperature (black dots, black regression line) across the study period at the Manu River in 2015. Mean day time temperatures are shown in orange and mean nighttime temperatures are shown in blue together with regression lines in respective colours. Ambient temperature was measured at the Pakitza meteorological station managed by the ‘Tropical Ecology, Assessment and Monitoring (TEAM) Network’, and supplied at 5‐min intervals. Two major flash floods on the Manu River took place in the study period, on 09th July 2015 and 10th Aug 2015, marking the distinction of breeding attempts by black skimmers shown in grey vertical bands and labelled A1, A2 and A3.

A meteorological station managed by the ‘Tropical Ecology, Assessment and Monitoring (TEAM) Network’ at Pakitza (11°56′45.83″ S, 71°16′47.71″ W) was situated 15 km from the Cocha Cashu Biological Station and on the bank of the Manu River. The range of air temperature measured during the study period was 15.34–36.27°C (mean = 24.21°C ± 3.83 Standard Deviation; SD), with mean daily temperatures increasing across the season (Figure A1).

### 2.2 Incubation behaviour

Nests along the Manu River were located by searching those parts of the beaches where birds were initially observed when passing slowly with a boat. After initial discovery, we visited nests to monitor nest content and deploy cameras and temperature loggers. The average visit frequency to beaches was 2.5 days (±0.9 SD).

To record incubation behaviour, we deployed 10 Bushnell Trophy Cam HD (Bushnell Corporation, Overland Park, KS, USA) and one Reconyx HC500 Hyperfire (Reconyx Inc., WI, USA) wildlife cameras 5 m from nests, facing the river. Infrared LED flash on the cameras allowed for night‐time observations. Cameras were programmed to take an image at 1‐min intervals, which was the highest time‐lapse frequency available for these camera models (motion‐activated pictures were not used in the present study due to large variability in the frequency of these images depending on the temperature of the surroundings).

Each camera monitored a single nest, due to the low nest density per beach (mean = 2 nests; maximum = 6 nests) and a relatively large distance between active nests (mean = 120.4 m; minimum = 13.6 m; Austad, 2016). To maximise the number of nests monitored, we therefore relocated cameras to a different nest approximately every 4 days. In total, 31 nests were monitored during the study.

### 2.3 Nest temperature

**FIGURE A2:**
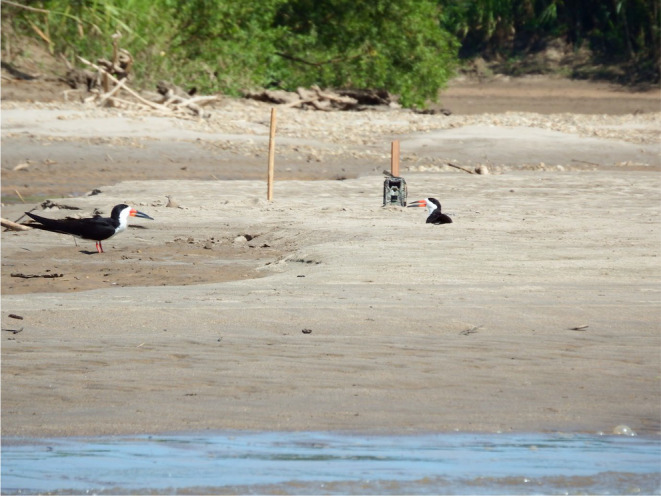
A black skimmer pair at a nest monitored with a trail camera, which is fastened to a stake so that the angle and position do not change despite the sand substrate. The *i*Button® measuring beach surface temperature in proximity to the nest was secured on top of a nail and tied with a string to the stake visible centrally in the image and allowed for relocating the *i*Button®. The female is incubating the nest (right) and the male is on the left.

To measure temperature in a sub‐sample of nests, we used DS1921G Thermochron *i*Buttons® with a recording frequency of 20 min and an accuracy of ±1°C and resolution of 0.5°C (Maxim Integrated Products 2015). *i*Buttons® were placed in the bottom of the nest, covered with a thin polyester mosquito netting and a thin layer of sand to avoid detection. Furthermore, the loggers were placed in small zip‐lock bags to prevent damage from humidity and precipitation (González del Pliego et al., 2016). The loggers were secured on long nails driven into the sand substrate, with the *i*Buttons® at the top, to reduce displacement (Cervencl et al., 2011; Hartman & Oring, 2006; Schneider & McWilliams, 2007). Due to the position of the loggers, we present the temperature measured as nest temperature and not the true incubation or egg temperature. For comparison, *i*Buttons® were also placed on the beach surface in proximity to nests (Figure A2), just covered by a thin layer of sand, but in the same netting and zip lock bag covering (Cervencl et al., 2011; Schneider & McWilliams, 2007). In order to re‐locate the beach surface *i*Buttons® we tied the nail with a string to a stake, while ensuring that the *i*Button® was outside the shade radius made by the stake.

In order to further test the effect of incubation on nest temperature, we compared nest temperature between two unattended and two attended nests containing the same number of eggs (two and three respectively) and on the same dates (18th to 24th July).

Air temperature was obtained from the meteorological station at Pakitza (refer to study area). These data had a 5‐min resolution and were used to test effect of ambient conditions on incubation behaviour and nest temperature.

### 2.4 Nest age

To control for any behavioural changes across the incubation period, we estimated the age of each nest as the number of days until hatching. We determined the approximate date when egg‐laying was complete by counting the number of eggs on each visit (Dinsmore, 2008). The number of nestlings was also counted during hatching, following the assumption that eggs hatched in the same sequence as they were laid (Grant & Hogg, 1976). In this way, the median number of days for an egg to hatch was calculated (*N* = 19 eggs from 9 nests). For three nests with the lowest visitation frequency and unrecorded hatching dates, an egg flotation model was used to predict nest age. The model was based on repeated measurements of egg float height and angle in water from 11 nests with observed hatching dates (Liebezeit et al., 2007; Mabee et al., 2006; Table A1).

### 2.5 Image analysis

Camera trap images were analysed for the incubation period, starting with the date when the largest clutch size was recorded until the first egg hatched per nest. Moreover, data were divided into three separate nesting attempts (A1, A2, A3), caused by the two flash floods. During A1 (29th June to 8th July) egg‐laying was not completed and therefore the attempt was not included in further analysis. A2 (13th July to 9th August) and A3 (19th August to 30th September, which was the end of the fieldwork period) were analysed separately to avoid pseudo‐replication. With only a small proportion of uniquely marked adults in the study population, we were unable to determine whether pairs chose the same beach or partner, though it is likely that at least some of the birds re‐nested after failure (Gochfeld, 1978; Groom, 2013).

A nest was included in the analysis only if a minimum of a complete 24‐h recording period was obtained. In cases of longer durations of recording, obtained for any particular nest, 24‐h periods of images were selected prior to viewing with up to four periods analysed per nest. Periods were selected to cover the largest range of nest age possible across the incubation period for any given nest. Image time was extracted from metadata using ExifTool‐10.07 (Harvey, 2016), through R (R Core Team, 2021). The same observer (MA) labelled all images.

**FIGURE 1:**
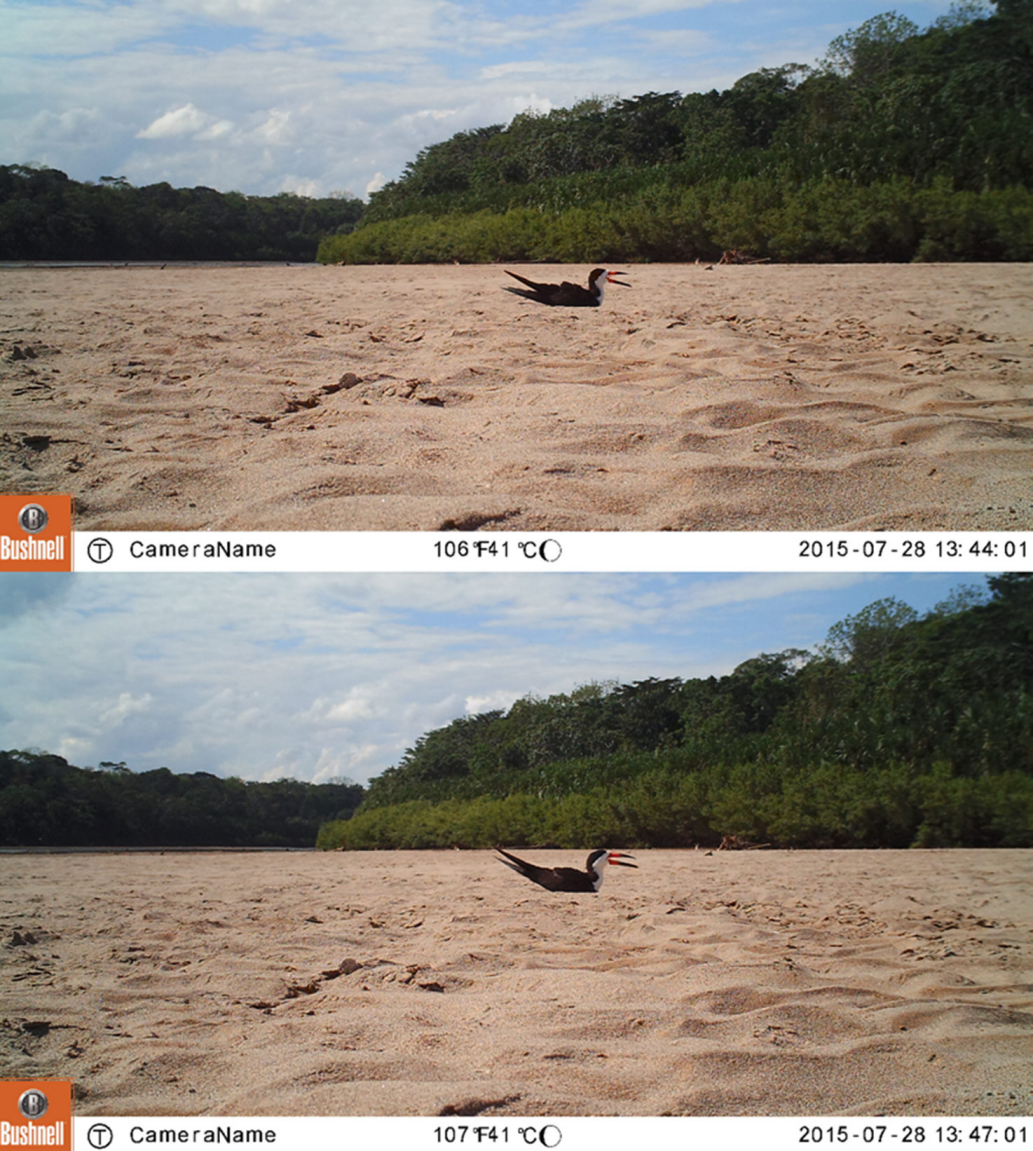
Black skimmers in camera trap images from the same nest at the Manu River, Peru. The female black skimmer is incubating in the top image, while the male is incubating in the lower image. The difference in body size, and shorter and thinner beak of the female is evident. In both images, the incubating bird is in the extended neck posture, while also gaping and raising scapulars.

For each image, the presence/absence of an incubating bird (attending the eggs but not necessarily maintaining contact between the brood patch and eggs) and the sex of the incubating bird were recorded. Male black skimmers were distinguished based on larger size, longer beaks and larger beak depth than females (Burger, 1981b; Quinn, 1990), reliable even for individuals viewed alone (Vieira et al., 2018a; Figure 1). Within each pair, the larger bird was always assumed to be the male, which is very likely taken a consistent size difference in measurements of the same subspecies (Vieira et al., 2018a). To further improve the reliability of sex determination within each pair, MA confirmed size differences by displaying multiple image panes next to each other in XnView 2.34, a free image viewing software. Even so, sex was not determined in all images, especially when the bird faced away from the camera without the head and bill showing or when image quality was poor due to fog.

An incubation posture where the neck was extended upwards was also recorded due to a potential thermoregulatory function (Bartholomew & Dawson, 1979; Figure 1). Furthermore, incubation bout length was defined as the duration of consecutive images with the same bird incubating and when a bird was present for only one image, a bout length of 1‐min was assigned. Incubation bouts with uncertain length due to images in which sex was not determined were excluded. Boats passing on the river were visible in image backgrounds. Because birds might alter their behaviour due to disturbance by passing boats, we excluded the respective incubation bout, irrespective of whether the incubating bird left the nest or not, together with the bout immediately before and after the affected bout.

### 2.6 Statistical analysis

RStudio 0.99.491–2021.09.1 (©2009–2021 RStudio, Inc.) run with R version 3.2.2‐4.05 (R Core Team, 2021) was used for statistical analysis, and graphical representations was carried out with ‘ggplot2’ package (Wickham, 2016).

We used Kendall's rank correlation to test the relationship between nest and beach surface temperatures and ambient air temperature. Two‐sided Mann–Whitney *U*‐tests for paired data with Bonferroni correction were used to test whether nest temperatures differed significantly from beach surface temperatures per hour. Additionally, we tested whether nest temperatures were higher or lower than beach surface temperatures per hour using one‐sided tests.

To determine whether incubation behaviour varies with temperature and throughout a 24‐h diel cycle, we used generalised linear‐mixed models (GLMMs). In order to model diel activity, the cosinor method was used, by which time of day is inserted into cosine functions as explanatory variables (Refinetti et al., 2007; Steen, 2017). Three different sets of functions were used to model potential activity wave forms, one with the fundamental period (24‐h period), the second with one added harmonic to the fundamental period (12‐h periodicity) and a third function with two added harmonics (8‐h periodicity) (Cornelissen, 2014). The three sets of cosine functions used separately as explanatory variables in GLMMS were as follows:

- Fundamental period (24‐h)
$$I\left(\cos\left(2*\mathrm{pi}*\text{Hour}/24\right)\right)+I\left(\sin\left(2*\mathrm{pi}*\text{Hour}/24\right)\right)$$
- Fundamental period with one added harmonic (12‐h periodicity)
$$\begin{array}{c} I\left(\cos\left(2*\mathrm{pi}*\text{Hour}/24\right)\right)+I\left(\sin\left(2*\mathrm{pi}*\text{Hour}/24\right)\right)\\ \;+I\left(\cos\left(2*2*\mathrm{pi}*\text{Hour}/24\right)\right)+I\left(\sin\left(2*2*\mathrm{pi}*\text{Hour}/24\right)\right) \end{array}$$
- Fundamental period with two added harmonics (8‐h periodicity)
$$\begin{array}{c} I\left(\cos\left(2*\mathrm{pi}*\text{Hour}/24\right)\right)+I\left(\sin\left(2*\mathrm{pi}*\text{Hour}/24\right)\right)\\ \;+I\left(\cos\left(2*2*\mathrm{pi}*\text{Hour}/24\right)\right)+I\left(\sin\left(2*2*\mathrm{pi}*\text{Hour}/24\right)\right)\\ \;+I\left(\cos\left(3*2*\mathrm{pi}*\text{Hour}/24\right)\;\right)+I\left(\sin\left(3*2*\mathrm{pi}*\text{Hour}/24\right)\;\right) \end{array}$$

First, we tested whether time of day as part of cosine functions, ambient temperature, nest age or seasonal progression (i.e. days after Julian date, taken as 1.06.2015) had effect on the presence/absence (scored as 1/0) of an incubating bird in each image. Second, we tested for effects on the probability of the incubating bird being the male in the pair (male = 1; female = 0). For this test, we excluded images in which an incubating bird was absent or in which the sex of the incubating bird was not identified. Third, we tested whether the same explanatory variables affected the presence/absence of the extended neck posture in incubating birds. Seasonal progression was included separately to nest age in A2 models, while it was not included at all in A3 models due to high correlation with nest age (−0.73). For all tests, temperature and time of day were not considered in the same models due to collinearity (VIF > 8.0), calculated using the package ‘car’ (Fox & Weisberg, 2019). For all the above GLMMs, we used a binomial family distribution and logit link (Bolker et al., 2009), and the default Laplace Approximation was applied using the ‘lme4‐package’ (Bates et al., 2015). When models failed to converge, the ‘bobyqa’ optimiser from the ‘minqa‐package’ (Bates et al., 2014) was included.

In a final set of GLMMs fit in the ‘glmmTMB‐package’ (Brooks et al., 2017), we tested the effects of incubating bird sex, time of day, nest age, days after Julian date in A2 and ambient temperature on log‐transformed incubation bout length, by using a Gaussian distribution and identity link.

Nest ID was included in all models as a random effect to account for the non‐independence of images from the same nest, and all four sets of GLMMs included a null model with only random effect. All the models are presented in Table A2.

We used the ‘AICcmodavg‐package’ (Mazerolle, 2023) to rank models and to select the most parsimonious model, that is, the one with as few predictor variables as possible among those with Δ AIC_c_ ≤ Δ2.0 (Arnold, 2010), while presenting the mean parameter estimates and their 95% confidence intervals (CI) using the Wald method in the ‘broom.mixed‐package’ (Bolker & Robinson, 2022). Additionally, in order to predict the mean response and 95% CIs across the range of each predictor variable in the selected models, we used the ‘predict’ function in the lme4 and glmmTMB packages. The ‘midline estimated statistic of rhythm’ (MESOR), which is a measure of the mean diel activity, was predicted using the respective intercept‐only models (Pita et al., 2011; Refinetti et al., 2007).

## 3 RESULTS

### 3.1 Incubation behaviour

We used annotated images that made up full 60‐min segments, resulting in behavioural classification in 38,460 images from nine nests on eight beaches for A2 models, and 33,840 images from eight nests on five beaches for A3. Incubating birds were identified as male or female in 95.47% and 97.38% of images with an incubating bird in A2 and A3 respectively.

Models with cosine functions representing diel effects on behaviour received more support in model ranking by AIC_c_ than models with temperature as the explanatory variable and the null models (Table 1; Tables A3 and A4).

**TABLE 1:** Generalised linear mixed model parameter estimates of the models with AIC_c_ ≤ Δ2.0, for different dependent variables of black skimmer incubation behaviour as classified from 1‐min time lapse images.

	A2					A3			
	Estimate	SE	Lwr 95% CI	Upr 95% CI		Estimate	SE	Lwr 95% CI	Upr 95% CI
*Response: Presence of incubating bird (logit link)*									
M_1.7_					M_1.7_				
(Intercept)	3.7455	0.1964	3.3605	4.1304	(Intercept)	4.3677	0.1544	4.0651	4.6704
I(cos(2 * pi * Hour/24))	−0.1439	0.0492	−0.2404	−0.0474	I(cos(2 * pi * Hour/24))	0.4191	0.0435	0.3339	0.5044
I(sin(2 * pi * Hour/24))	0.1539	0.0378	0.0797	0.2281	I(sin(2 * pi * Hour/24))	0.3172	0.0345	0.2496	0.3848
I(cos(2 * 2 * pi * Hour/24))	0.6159	0.0432	0.5312	0.7005	I(cos(2 * 2 * pi * Hour/24))	0.4309	0.0386	0.3552	0.5066
I(sin(2 * 2 * pi * Hour/24))	−0.0155	0.0412	−0.0963	0.0653	I(sin(2 * 2 * pi * Hour/24))	−0.0412	0.0378	−0.1153	0.0329
I(cos(3 * 2 * pi * Hour/24))	0.1711	0.0419	0.0889	0.2533	I(cos(3 * 2 * pi * Hour/24))	0.1215	0.0371	0.0488	0.1943
I(sin(3 * 2 * pi * Hour/24))	−0.2855	0.0418	−0.3673	−0.2037	I(sin(3 * 2 * pi * Hour/24))	−0.1464	0.0376	−0.2200	−0.0728
Nest Age	−0.0290	0.0073	−0.0432	−0.0147	Nest Age	−0.1138	0.0078	−0.1291	−0.0985
*Response: Presence of male incubating (logit link)*									
M_2.7_					M_2.7_				
(Intercept)	−0.0105	0.0643	−0.1366	0.1155	(Intercept)	0.3037	0.1246	0.0595	0.5478
I(cos(2 * pi * Hour/24))	−0.0720	0.0169	−0.1051	−0.0389	I(cos(2 * pi * Hour/24))	−0.1177	0.0175	−0.1521	−0.0833
I(sin(2 * pi * Hour/24))	−0.0354	0.0169	−0.0684	−0.0023	I(sin(2 * pi * Hour/24))	0.0113	0.0171	−0.0222	0.0447
I(cos(2 * 2 * pi * Hour/24))	0.0107	0.0169	−0.0225	0.0439	I(cos(2 * 2 * pi * Hour/24))	−0.0489	0.0174	−0.0829	−0.0148
I(sin(2 * 2 * pi * Hour/24))	0.1006	0.0168	0.0676	0.1336	I(sin(2 * 2 * pi * Hour/24))	0.0173	0.0173	−0.0166	0.0511
I(cos(3 * 2 * pi * Hour/24))	0.2025	0.0168	0.1695	0.2355	I(cos(3 * 2 * pi * Hour/24))	0.1415	0.0173	0.1076	0.1753
I(sin(3 * 2 * pi * Hour/24))	0.0409	0.0167	0.0081	0.0737	I(sin(3 * 2 * pi * Hour/24))	−0.0093	0.0174	−0.0434	0.0247
Nest age	−0.0286	0.0028	−0.0342	−0.0231	Nest Age	−0.0618	0.0046	−0.0708	−0.0528
*Response: Extended neck posture (logit link)*									
M_3.10_					M_3.4_				
(Intercept)	−8.6044	0.3290	−9.2492	−7.9595	(Intercept)	−2.3801	0.1420	−2.6585	−2.1018
I(cos(2 * pi * Hour/24))	−1.0164	0.0283	−1.0718	−0.9609	I(cos(2 * pi * Hour/24))	−1.6888	0.0337	−1.7549	−1.6226
I(sin(2 * pi * Hour/24))	−0.2966	0.0326	−0.3606	−0.2326	I(sin(2 * pi * Hour/24))	−0.6865	0.0387	−0.7625	−0.6106
I(cos(2 * 2 * pi * Hour/24))	0.5699	0.0314	0.5085	0.6314	I(cos(2 * 2 * pi * Hour/24))	0.6863	0.0348	0.6182	0.7544
I(sin(2 * 2 * pi * Hour/24))	0.0213	0.0291	−0.0357	0.0783	I(sin(2 * 2 * pi * Hour/24))	−0.1250	0.0363	−0.1961	−0.0539
I(cos(3 * 2 * pi * Hour/24))	−0.2384	0.0279	−0.2931	−0.1836	I(cos(3 * 2 * pi * Hour/24))	0.1281	0.0307	0.0679	0.1883
I(sin(3 * 2 * pi * Hour/24))	0.2681	0.0287	0.2118	0.3244	I(sin(3 * 2 * pi * Hour/24))	0.0978	0.0302	0.0385	0.1571
Days after Julian day	0.1015	0.0048	0.0921	0.1108					
					M_3.7_				
					(Intercept)	−2.2649	0.1554	−2.5693	−1.9604
					I(cos(2 * pi * Hour/24))	−1.6909	0.0338	−1.7571	−1.6247
					I(sin(2 * pi * Hour/24))	−0.6858	0.0387	−0.7617	−0.6098
					I(cos(2 * 2 * pi * Hour/24))	0.6878	0.0348	0.6196	0.7560
					I(sin(2 * 2 * pi * Hour/24))	−0.1259	0.0363	−0.1970	−0.0548
					I(cos(3 * 2 * pi * Hour/24))	0.1267	0.0307	0.0665	0.1869
					I(sin(3 * 2 * pi * Hour/24))	0.0988	0.0303	0.0395	0.1581
					Nest age	−0.0111	0.0065	−0.0238	0.0016
*Response: log*_*10*_ *(Incubation bout length) (identity link)*									
M_4.19_					M_4.13_				
(Intercept)	1.7602	0.1714	1.4243	2.0962	(Intercept)	1.3366	0.0445	1.2494	1.4238
I(cos(2 * pi * Hour/24))	0.0882	0.0163	0.0563	0.1200	I(cos(2 * pi * Hour/24))	0.1995	0.0167	0.1667	0.2323
I(sin(2 * pi * Hour/24))	0.0870	0.0165	0.0547	0.1192	I(sin(2 * pi * Hour/24))	0.0798	0.0163	0.0479	0.1118
I(cos(2 * 2 * pi * Hour/24))	0.0059	0.0159	−0.0252	0.0371	I(cos(2 * 2 * pi * Hour/24))	−0.0065	0.0157	−0.0373	0.0244
I(sin(2 * 2 * pi * Hour/24))	0.0045	0.0167	−0.0282	0.0373	I(sin(2 * 2 * pi * Hour/24))	−0.0097	0.0173	−0.0435	0.0242
I(cos(3 * 2 * pi * Hour/24))	0.0609	0.0163	0.0290	0.0928	I(cos(3 * 2 * pi * Hour/24))	0.0348	0.0161	0.0031	0.0664
I(sin(3 * 2 * pi * Hour/24))	−0.0492	0.0158	−0.0802	−0.0182	I(sin(3 * 2 * pi * Hour/24))	−0.0535	0.0161	−0.0850	−0.0220
MaleFemale: male	−0.0922	0.0221	−0.1355	−0.0488	MaleFemale: male	−0.0490	0.0223	−0.0928	−0.0053
Days after Julian day	−0.0093	0.0028	−0.0148	−0.0038	Nest age	−0.0203	0.0034	−0.0270	−0.0137

*Note*: Two distinct breeding attempts were analysed out of a total of three which occurred during study season, the second (A2) is presented on the left and the third (A3) on the right. Each model included Nest ID as a random effect. Model numbers are included for each model so reference can be made to AIC_c_ ranking tables in Table A3.

**FIGURE 2:**
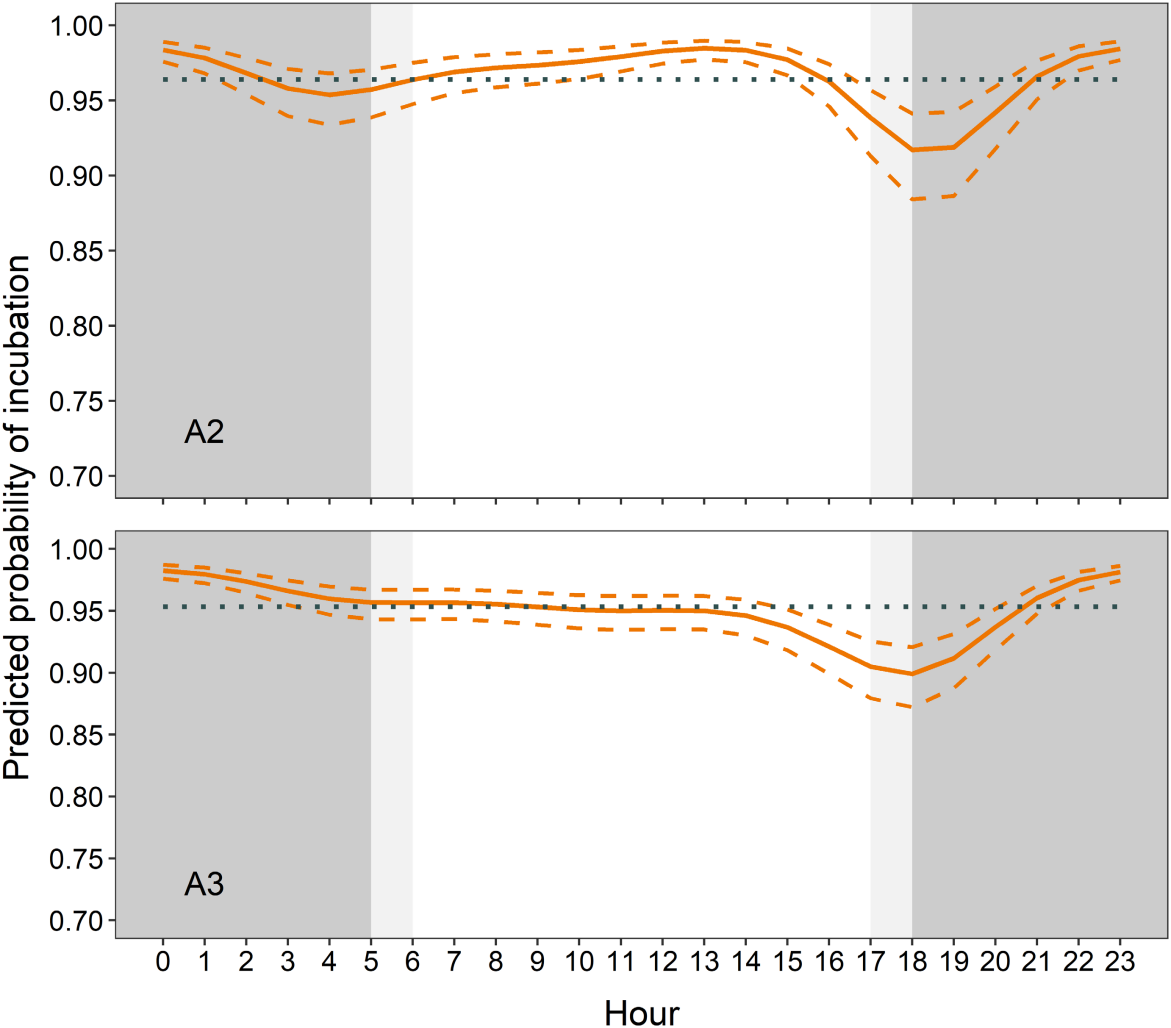
Black skimmer mean predicted (orange solid line) and 95% CI (orange dashed lines) probability of incubation across the diel cycle deriving from generalised linear mixed models on the presence/absence of an incubating bird in time‐lapse images collected along the River Manu, Peru. Cosine functions were used to test for the effect of time and diel patterns and parameter estimates of the models are presented in Table 1 (A2: M_1.7_; A3: M_1.7_). The second breeding attempt (A2) is presented on top (*N* = 38,460 images), and the third breeding attempt (A3) is presented on the bottom (*N* = 33,840 images). The *y*‐axis is scaled at 0.7 to 1.0. The ‘midline estimated statistic of rhythm’ (MESOR), is shown with the dotted horizontal line. Dark grey background represents nighttime and light grey represents twilights.

Mean diel egg attendance by adult black skimmers (MESOR) was predicted at 0.964 (95% CI 0.948–0.975) in A2 and 0.953 (95% CI 0.942–0.963) in A3. However, in both breeding attempts, incubation probability decreased significantly (CIs did not overlap MESOR) between 17:00 and 20:00 h (Figure 2). In A2, incubation probability was significantly higher between 11:00 and 15:00 h (Figure 2). Incubation probability increased closer to hatching (A2: −0.030, 95% CI −0.0432 to −0.0147; A3: −0.114, 95% CI −0.129 to −0.099; Table 1).

**FIGURE 3:**
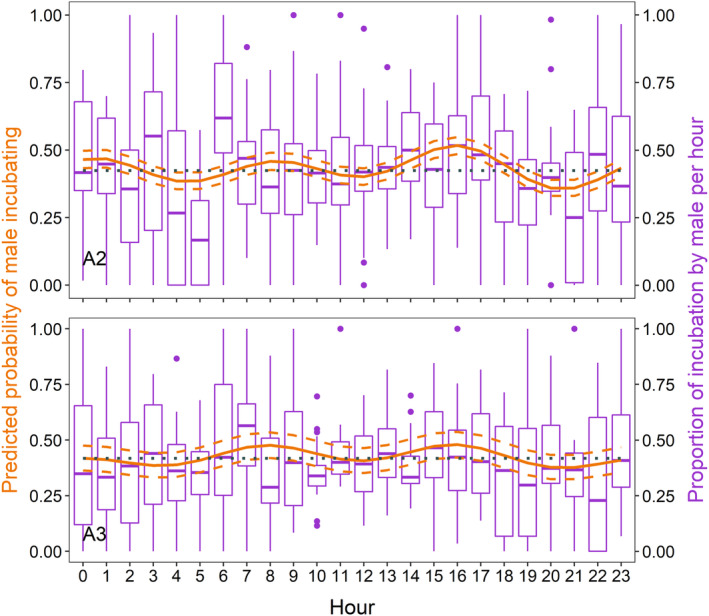
Mean predicted (orange solid line) and 95% CI (orange dashed lines) probability of incubation by male black skimmers across the diel cycle derived from generalised linear mixed models on the presence/absence of males incubating in time‐lapse images. The ‘midline estimated statistic of rhythm’ (MESOR), is shown with the dotted horizontal line. Boxplots (purple) show the proportion male incubation per hour. The second breeding attempt (A2) is presented on the top (*N* = 30,101 images), and the third breeding attempt (A3) is presented on the bottom (*N* = 28,656 images), while the parameter estimates for the models on which prediction was made are presented in Table 1 (A2: M_2.7_, A3: M_2.7_).

The probability of incubation by males was modelled on 30,101 and 28,656 images in A2 and A3, following the removal of hours in which the sex of the incubating bird was not determined. Males were less likely to incubate compared to the females with MESOR predicted at 0.424 (95% CI 0.394–0.455) in A2 and 0.419 (95% CI 0.371–0.468) in A3. The probability of incubation by males was highest at 08:00 and 16:00 h in both breeding attempts, but during the middle of the day (10:00 to 14:00 h) male contribution varied the least compared to the rest of the diel cycle (Figure 3). The probability of incubation by males also increased closer to hatching (A2: −0.029, 95% CI −0.034 to −0.023; A3: −0.062, 95% CI −0.071 to −0.053; Table 1).

**FIGURE 4:**
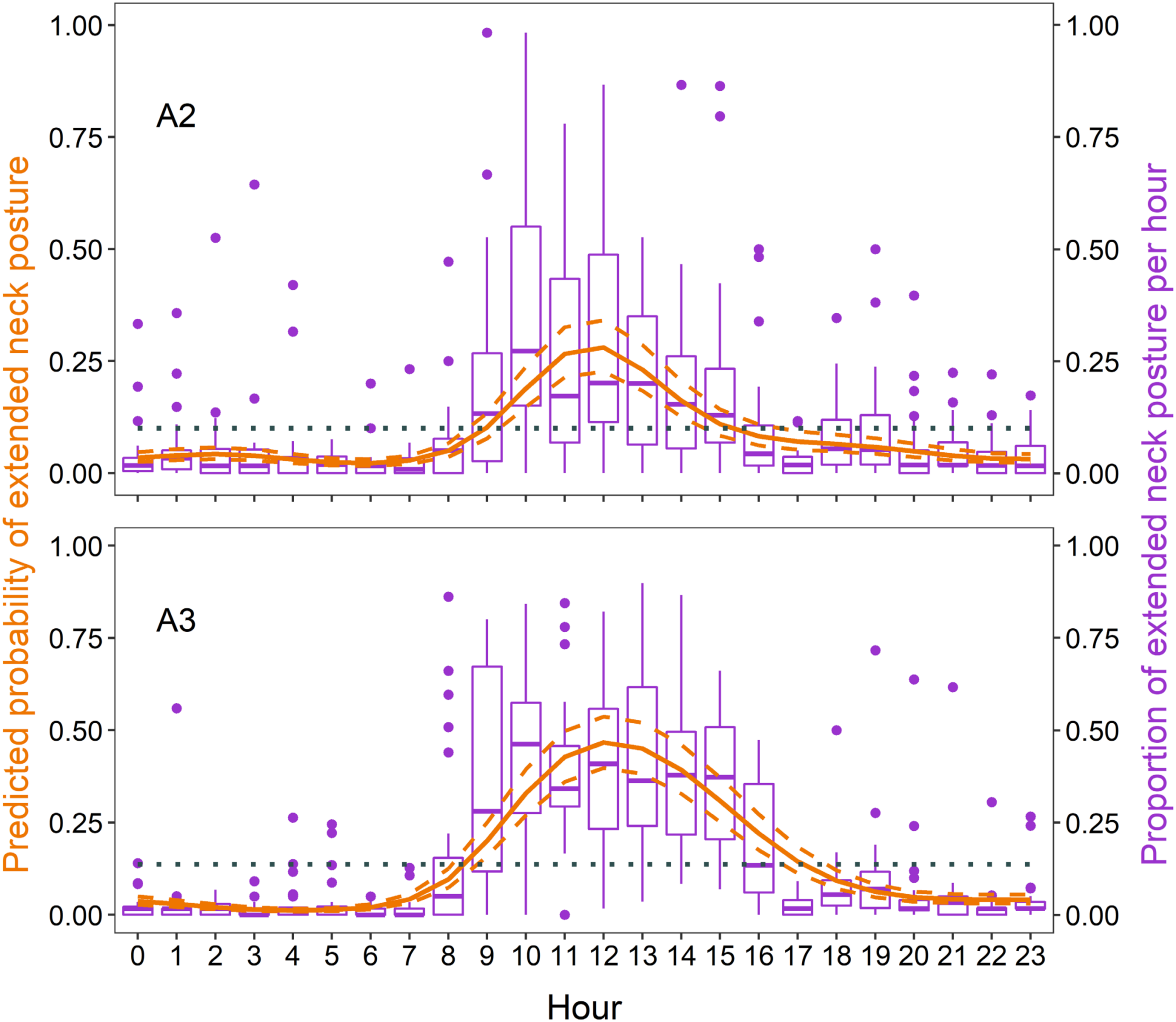
Mean predicted (orange solid line) and 95% CI (orange dashed lines) probability of an extended neck posture in incubating black skimmers across the diel cycle, derived from generalised linear mixed models on the presence/absence of the posture in time‐lapse images. The ‘midline estimated statistic of rhythm’ (MESOR), is shown with the dotted horizontal line. Boxplots (purple) show the proportion of the incubating birds with an extended neck posture per hour. The second breeding attempt (A2) is presented on the top (*N* = 37,060 images), and the third breeding attempt (A3) is presented on the bottom (*N* = 32,115 images), while the parameter estimates for the models on which prediction was made are presented in Table 1 (A2: M_3.10_, A3: M_3.4_).

After removing images in which there was no incubating bird, model testing for effects on posture was run on 37,060 images in A2 and 32,115 in A3. The probability of extended neck posture MESOR was predicted at 0.100 (95% CI 0.078–0.128) in A2 and 0.137 (95% CI 0.112–0.166) in A3. The probability of the posture increased significantly with the progression of the season in A2 (0.101, 95% CI 0.092–0.111; Table 1) but we did not find evidence for variation across days in A3 (Table 1). Across the diel cycle, the posture peaked at 12:00 h (Figure 4). While ambient temperature alone explained less of the variation in the response compared to the best ranking models that included the 8‐h periodicity cosinor functions (Tables A2 and A3), the posture also increased with increasing temperature (A2: 0.259, 95% CI 0.249–0.268; A3: 0.306, 95% CI 0.297–0.315; Table A4).

**FIGURE 5:**
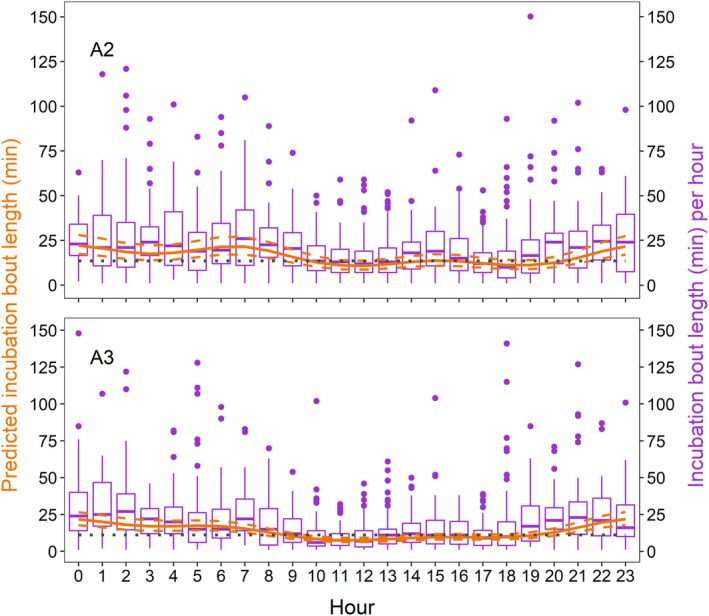
Predicted (orange solid line) and 95% CI (orange dashed lines) bout length by incubating black skimmers, derived from generalised linear mixed models (A2: M_4.19_, A3: M_4.13_; Table 1). Bout length (min) was normalised by log10 transformation, but predicted response is back transformed. Incubation bouts were obtained from in 1‐min frequency time‐lapse images, in which the presence of the sexually dimorphic male and female were recorded per image. Cosine functions were used to test for the effect of time and diel patterns. The ‘midline estimated statistic of rhythm’ (MESOR), is shown with the dotted horizontal line. Boxplots (purple) show the length of bouts per hour. The second breeding attempt (A2) is presented on top (*N* = 1449 bouts), and the third breeding attempt (A3) is presented on the bottom (*N* = 1603 bouts).

In A2, 1449 bouts and in A3, 1603 bouts were retained for analysis. The mean incubation bout length was 20.72 min (±17.74 SD) in A2 and 17.67 min in A3 (±17.76 SD), with maximum lengths recorded at night of up to 150 min (Figure 5). Male black skimmers were found to incubate for significantly shorter bouts than females (A2: −0.092, 95% CI −0.136 to −0.049; A3: −0.049, 95% CI −0.093 to −0.005; Table 1). This effect resulted in a predicted bout length of 13.93 min (95% CI 11.09–17.50) and 17.23 min (95% CI 13.74–21.60) in A2 and 12.14 min (95% CI 10.04–14.67) and 13.59 min (95% CI 11.23–16.38) in A3, for males and females respectively. Black skimmers incubated for shorter bouts in the middle of the day, an effect that was significant compared to MESOR in A3 (Figure 5). Incubation bout length also changed with seasonal progression in A2 (−0.009, 95% CI −0.015 to −0.004; Table 1) and nest age in A3 (−0.020, 95% CI −0.027 to −0.014).

### 3.2 Nest temperature

**FIGURE 6:**
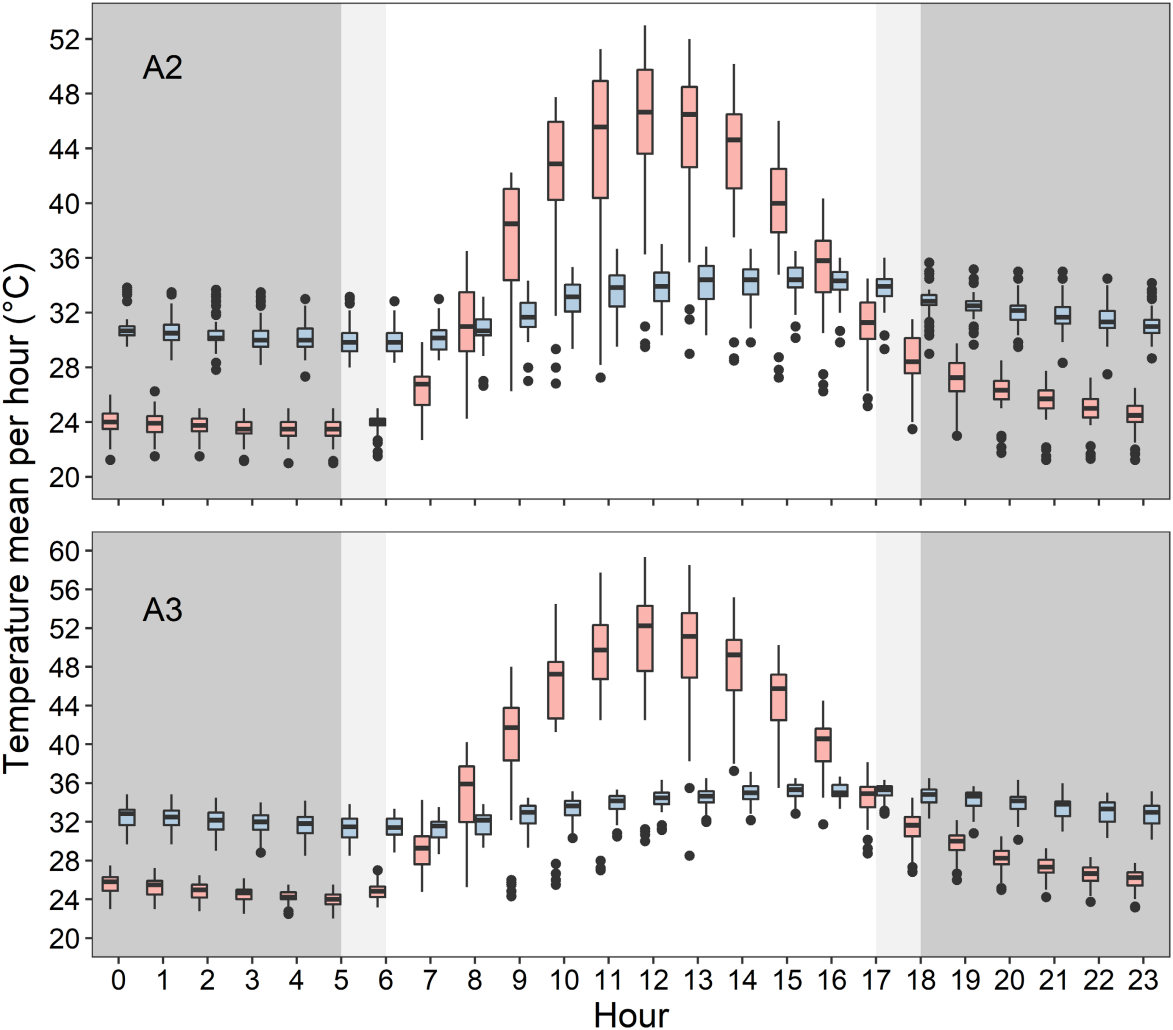
Black skimmer nest temperature (light blue boxplots) and respective beach surface temperature (light red boxplots) per hour, measured using *i*Button® temperature loggers on beaches along the Manu River, Peru. Three nests were included for the second breeding attempt (A2), top, and five nests were included for the third breeding attempt (A3), bottom. Dark grey background represents nighttime and light grey represents twilights.

We obtained nest temperature with comparative beach surface measurements during the incubation period for three nests in A2 (*N* = 802 h) and five nests in A3 (*N* = 1209 h). Nest temperature was less affected by ambient temperature (A2: *r* = .568, *p* < .001; A3: *r* = .462, *p* < .001) than beach surface temperature (A2: *r* = .7885, *p* < .001; A3: *r* = .774, *p* < .001). On average, nest temperature was 6.37 ± 1.84 and 6.28 ± 1.78°C warmer at night than beach surface temperature (*p* < .001), and 6.40 ± 6.25 and 9.74 ± 7.53°C cooler during the day (*p* < .001) for A2 and A3 respectively (Figure 6). However, beach surface and nest temperature did not differ significantly in two‐sided tests at 08:00 h in A2 (*p* = .42), at 16:00 h in A2 (*p* = .08) or at 17:00 h (*p* = .06) in A3 (Figure 6; Table A5).

Nest temperature differed between two attended and two unattended nests (*p* < .001), where the range of temperatures measured in the attended nests was 26.42–37.17°C, while in unattended nests it was 22.17–43.50°C.

## 4 DISCUSSION

Black skimmer pairs attended nests almost continuously day and night, and their incubation effort kept nest temperatures relatively constant irrespective of ambient and beach surface temperature fluctuations. However, incubation bouts were shorter and extended neck postures were more frequent around midday, suggesting thermally challenging conditions at higher ambient temperatures. The near‐continuous incubation by black skimmers clearly buffers for temperature extremes on the unvegetated and exposed beaches on which they nest. During the day, nest temperatures were approx. 36°C, while temperatures regularly exceeded 50°C on the unshaded beach surface. At night, nest temperature was around 28°C, whereas the beach surface was much cooler and sometimes lower than 24°C. Avian eggs have a relatively narrow range of temperature tolerance (DuRant et al., 2013) and although embryos themselves show behavioural and physiological plasticity in response to variation in temperature (Du & Shine, 2015), most birds attend nests to mitigate unfavourable thermal conditions. Although specific knowledge on thermal tolerance of black skimmer eggs is lacking, it is likely that nest attendance prevented potential lethal and sub‐lethal effects, which generally start above 40°C in birds (DuRant et al., 2013; Grant, 1982; Webb, 1987). At night, high incubation probability was likely a means to avoid suspension of embryo development that occurs below 26°C (DuRant et al., 2013). Some differences between beach surface and nest temperature may be attributed to favourable microclimate conditions favoured during nest site selection, rather than nest attendance alone. Some differences might also be attributed to the inherent limitations in *i*Button® placement, where those in nests were placed in the bottom of a shallow scrape containing eggs, while the control *i*Buttons® were placed on the beach surface in the proximity of nests. However, a comparison between two active and two unattended nests with an equal number of eggs, found active nests to have a significantly narrower range of temperatures. Moreover, similar incubation behaviour and function have been observed in other species nesting in environments with thermal extremes across the diel cycle, where adults keep nests cooler during the day and warmer during the night (AlRashidi, 2016; AlRashidi & Shobrak, 2015; Carroll et al., 2018; Clauser & McRae, 2017; Mougeot et al., 2014; Sullivan et al., 2020).

Black skimmer incubation probability was lowest at dusk (18:00 h), when ground temperature was similar to nest temperature. This is consistent with other studies showing that ground nesting species have decreased incubation probabilities in diel periods when ground temperature is similar to optimal incubation temperature (Mougeot et al., 2014). Since black skimmers did not portray a lower incubation probability during similar conditions in the morning (08:00 h), we interpret this result as a strategy to take advantage of the overlap between favourable thermal and foraging conditions. This is similar to previous observations of Saunders's Tern *Sternula saundersi* (AlRashidi & Shobrak, 2015). Indeed, black skimmers are predominantly crepuscular and nocturnal foragers (Grant & Hogg, 1976; Rojas et al., 1997), implying that the foraging and thermal optima only overlapped around dusk. Along the River Manu they forage during the day at lower frequency and only when provisioning chicks (Groom, 1992, M. Austad, personal observation). The longer incubation bouts recorded at night are expected to allow the non‐incubating partner to forage further away from the nest site. Black skimmers breeding along the Manu river have a foraging range of at least 15 km (Davenport et al., 2016; Vieira et al., 2018b), but further research is required on the species' foraging movements.

We found highly shared biparental incubation in Amazonian black skimmers across the diel cycle. Overall, males were found to incubate slightly less than females, complementing results from the Brazilian Amazon using only diurnal observations (Schuchmann et al., 2022). Incubation was shared most equally between 10:00 to 14:00 h, in contrast to larger variations in contribution during other parts of the diel period (Figure 3). It follows that incubation bout length also decreased in the middle of the day as partners swapped more frequently. This underlines the importance of biparental care in black skimmers to be able to regulate nest temperatures while simultaneously preventing overheating of their own bodies. These results are in line with other studies showing higher synchronisation between partners and shorter incubation bouts during midday (AlRashidi et al., 2010; Sullivan et al., 2020; Ward, 1990) particularly where water bodies are available (Amat & Masero, 2004; Hand et al., 1981).

**FIGURE A3:**
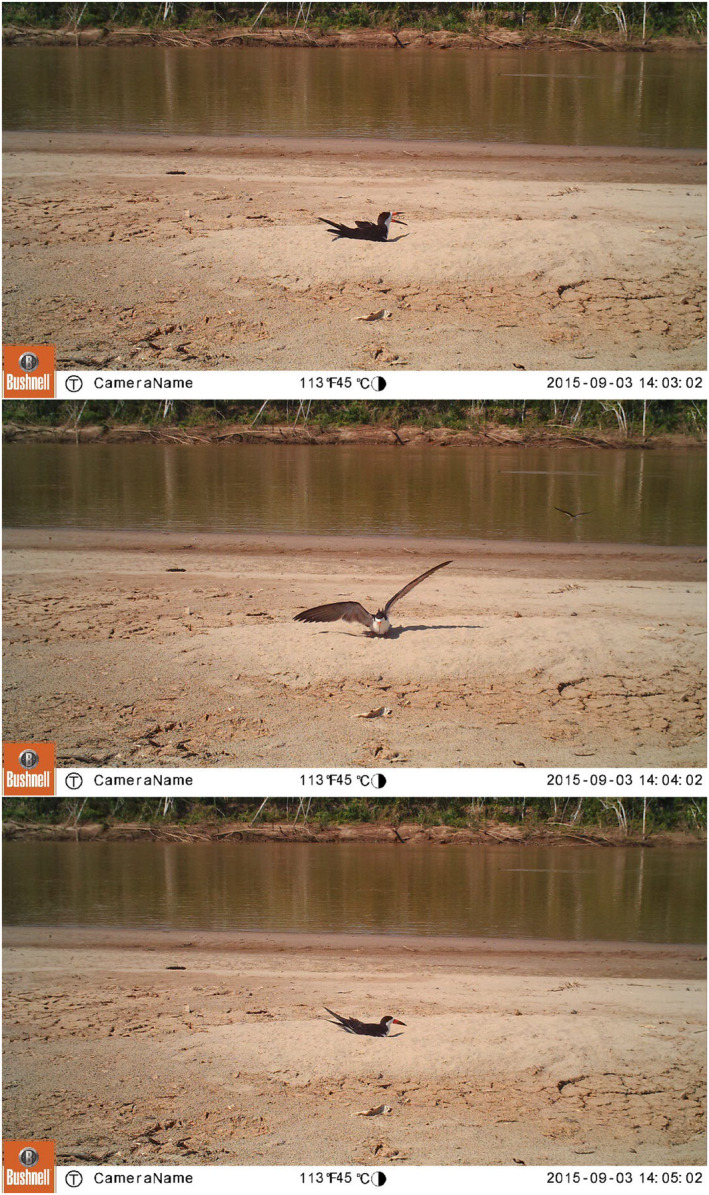
Black skimmers in time‐lapse images from the same nest at the Manu River, Peru. The male black skimmer is incubating in the top image, in an extended neck posture. In the central image, the pair exchange, and the male is seen to ‘belly‐soak’ in the river (right side of image). The female resumes incubation in the lower image, not yet taking on the extended neck posture.

Black skimmers at Manu were observed to carry out ‘belly‐soaking’ in the river (Groom, 2013, M. Austad, personal observation, Figure A3). Belly soaking behaviour is likely linked to the shorter incubation bouts at midday. This behaviour was documented to be a heat dissipating mechanism for thermally stressed incubating adult Kentish plovers *Charadrius alexandrinus*, but it also functions to cool down eggs (Amat & Masero, 2004). Flight produces endogenous heat, making belly soaking behaviour counterproductive if birds have to fly longer distances between their nest and water (Amat & Masero, 2004). Therefore, it is likely that the propensity of black skimmers to nest close to water (Zarza et al., 2013) might have thermoregulatory functions beyond nest microclimate (Davenport et al., 2016).

The posture of incubating birds varied across the diel cycle and with seasonal progression. Black skimmers were most likely to show an extended neck posture during midday, when ambient temperature often peaks. Birds can enlarge surface area and expose the head to higher rates of convection by extending their neck (Bartholomew & Dawson, 1979). The posture is therefore associated with less distinctive methods of thermoregulation such as gaping, wing drooping and ptiloerection of dorsal feathers (Grant & Hogg, 1976), not always visible in camera trap images. The relatively low MESOR predicted for this posture might indicate the potential of further behavioural plasticity in response to even higher temperatures. However, occasionally incubating birds were already close to constantly making use of heat dissipation mechanisms, especially at midday. Although we were not able to quantify the effect of local wind on incubation behaviour in this study, even a light breeze has been shown to increase convective cooling from birds especially when they raise feathers and neck (Bartholomew & Dawson, 1979; Oswald & Arnold, 2012). These effects are even more beneficial for black plumages such as the dorsal parts of the black skimmer, leading to lower energy gain from solar radiation (Walsberg et al., 1978). In the Amazon, afternoon river breezes are driven by differences in river and land temperatures (Pereira de Oliveira & Fitzjarrald, 1993; Santos et al., 2019), which might reduce the impact of high temperatures on incubating birds but merits further research focusing on river size, water levels and nest placement.

Female black skimmers had longer incubation bouts than males, possibly indicating different thermal tolerances and efficiency of cooling mechanisms due to size dimorphism. Smaller size in females could mean that they could cool down more efficiently during shorter recesses than the larger males (Ward, 1990). Different thermoregulatory needs arising from size dimorphism have been shown to drive sexual habitat segregation in other endotherms (Alonso et al., 2016), but the potential influence on parental care is poorly known (Blanckenhorn, 2005). Thermal imaging could reveal different heat loss rates between black skimmer sexes and during different postures (McCafferty, 2013).

Through the use of camera traps with infrared LED flash, we were able to determine several behaviours across the diel cycle in black skimmers, and since the camera models used had infrared illumination rather than white light flash, we decreased disturbance for birds we monitored (Edney & Wood, 2021). Skimmers can see well in low light situations as they have a higher rod to cone ratio which could make them sensitive to white light flashes (Rojas et al., 1997). However, further classification of behaviour was constrained by the angle and distance of cameras from the nest. For example, whether birds were panting was not visible when they faced away from the camera. Moreover, to what extent birds shaded the eggs by standing over them rather than maintaining contact with the incubation patch (AlRashidi, 2016; Clauser & McRae, 2017; Ward, 1990), was not possible to determine in the present study. The use of 1‐min time‐lapse images meant that certain behaviours such as any incubation bouts shorter than 1‐min or the same bird leaving the nest within a minute might have gone undetected. However, mean bout length in this study was very similar to one using video recordings (Schuchmann et al., 2022), showing that time‐lapse cameras can be as reliable but probably more efficient than video recorders.

Apart from diel effects, black skimmers incubation behaviour changed with nest age and seasonal progression. The increased incubation attentiveness closer to hatching is expected due to parental investment theory but also potential changes in embryo thermal tolerance (Webb, 1987). Comparing the two breeding attempts, nest attendance at midday in A3 was lower than in A2 and incubation bouts were shorter. Birds might have been unable to make similar incubation effort because of reduced body condition across the season, especially females having made replacement clutches (DuRant et al., 2013; Kalmbach et al., 2004). On the other hand, temperatures increased during each attempt and were also higher in A3 compared to A2, which might in part explain these observations due to the need for more frequent belly soaking trips by incubating adults. We are unable to determine whether or how close black skimmers were to thermal limits while carrying out incubation duties but encourage further studies, using similar time‐lapse methods, to make comparisons across wider temperature conditions. Rising temperatures as caused by climate change is an increasing threat to incubation success for other species (Clauser & McRae, 2017; Cook et al., 2020). In the study period, in line with the gradual increase in mean air temperatures in Amazonia, there was a positive anomaly of around 0.5°C compared to the 1947–2017 mean, but record temperatures were reached during the El Niño event the following year (Marengo et al., 2018). Extreme hydrological events in the Amazon have also increased in frequency during the last decades, while droughts might prevail towards the end of the century (Zulkafli et al., 2016). Altered flooding regimes may increase flight distances for beach nesting birds when belly soaking, which in turn might make them more vulnerable to increasing temperatures and even nest desertion (Amat & Masero, 2004). In coastal breeding areas, climate change‐driven extreme weather events and sea level rise are already having a considerable impact on black skimmer nesting habitat, leading to a need for active conservation measures to offer alternative breeding locations (Coburn et al., 2001; Maslo et al., 2018; Tattoni et al., 2021). We recommend including the need for short and safe flight paths to water into these initiatives so that continuous incubation is not disrupted by long belly soaking recesses.

## 5 CONCLUSION

In conclusion, black skimmers use highly synchronised biparental care, make use of postures with thermoregulatory functions and shorten incubation bouts during midday when temperatures are highest. Moreover, they take advantage of optimal foraging conditions during a period of low incubation demand caused by an overlap in ground and nest temperatures. Studies are needed to understand how close sandbank nesting species may be to thermally tolerable maxima during incubation. Conservation efforts will be increasingly needed to counteract anthropogenic impacts on shore nesting birds, and such measures should incorporate thermoregulatory requirements in a warming world.

## AUTHOR CONTRIBUTIONS

**Martin Austad:** Conceptualization (equal); data curation (lead); formal analysis (lead); investigation (lead); methodology (lead); visualization (lead); writing – original draft (lead); writing – review and editing (equal). **Jørgen Sand Sæbø:** Conceptualization (supporting); data curation (equal); formal analysis (supporting); investigation (supporting); methodology (supporting); validation (supporting); visualization (supporting); writing – original draft (supporting); writing – review and editing (supporting). **Ronny Steen:** Conceptualization (equal); data curation (supporting); formal analysis (equal); investigation (supporting); methodology (equal); supervision (equal); validation (equal); visualization (supporting); writing – original draft (supporting); writing – review and editing (equal). **Katharine S. Goodenough:** Conceptualization (supporting); investigation (supporting); methodology (supporting); validation (supporting); writing – review and editing (equal). **Lisa Davenport:** Conceptualization (supporting); methodology (supporting); project administration (supporting); validation (supporting); writing – review and editing (equal). **Torbjørn Haugaasen:** Conceptualization (equal); data curation (supporting); formal analysis (supporting); funding acquisition (lead); investigation (supporting); methodology (supporting); project administration (lead); resources (lead); supervision (equal); visualization (supporting); writing – original draft (supporting); writing – review and editing (equal).

## CONFLICT OF INTEREST STATEMENT

The authors declare that they have no conflict of interest.

## Data Availability

All data used for this study have been deposited to Zenodo open repository on https://doi.org/10.5281/zenodo.8329522 for breeding attempt two (A2) and https://doi.org/10.5281/zenodo.8329543 for breeding attempt three (A3).

## APPENDIX 1

**TABLE A1:** Parameter estimates of egg flotation model used for prediction of days until hatching. The model is based on 11 nests with known hatching dates (mean visit frequency between 1.8 and 3.3 per nest).

Variable	Estimate	SE	df	*p*
(Intercept)	25.369	1.271	10	<.001
Angle	−0.175	0.021	4	.001
Flotation height	−1.221	0.392	4	.036

**TABLE A2:** Explanatory variables for all generalised linear mixed models with different dependent variables of black skimmer incubation behaviour as classified from 1‐min time lapse images. Two distinct breeding attempts were analysed separately (A2 and A3). Each model included Nest ID as a random effect, the null model always being denoted by M_.1_. The models as ranked by AIC_c_ are presented in Table A3 with the same model numbers for reference.

Model	Explanatory variable(s)	Random effect
**Breeding Attempt A2**		
*Response: Presence of incubating bird (logit link)*		
M_1.1_	(Null model)	Nest ID
M_1.2_	Fundamental period (24‐h)	Nest ID
M_1.3_	Fundamental period with one added harmonic (12‐h periodicity)	Nest ID
M_1.4_	Fundamental period with two added harmonics (8‐h periodicity)	Nest ID
M_1.5_	Fundamental period (24‐h) + Nest age	Nest ID
M_1.6_	Fundamental period with one added harmonic (12‐h periodicity) + Nest Age	Nest ID
M_1.7_	Fundamental period with two added harmonics (8‐h periodicity) + Nest Age	Nest ID
M_1.8_	Fundamental period (24‐h) + Days after Julian day	Nest ID
M_1.9_	Fundamental period with one added harmonic (12‐h periodicity) + Days after Julian day	Nest ID
M_1.10_	Fundamental period with two added harmonics (8‐h periodicity) + Days after Julian day	Nest ID
M_1.11_	Nest age + Ambient temperature	Nest ID
M_1.12_	Nest age	Nest ID
M_1.13_	Ambient temperature	Nest ID
M_1.14_	Days after Julian day	Nest ID
M_1.15_	Days after Julian day + Ambient temperature	Nest ID
*Response: Presence of male incubating (logit link)*		
M_2.1_	(Null model)	Nest ID
M_2.2_	Fundamental period (24‐h)	Nest ID
M_2.3_	Fundamental period with one added harmonic (12‐h periodicity)	Nest ID
M_2.4_	Fundamental period with two added harmonics (8‐h periodicity)	Nest ID
M_2.5_	Fundamental period (24‐h) + Nest age	Nest ID
M_2.6_	Fundamental period with one added harmonic (12‐h periodicity) + Nest Age	Nest ID
M_2.7_	Fundamental period with two added harmonics (8‐h periodicity) + Nest Age	Nest ID
M_2.8_	Fundamental period (24‐h) + Days after Julian day	Nest ID
M_2.9_	Fundamental period with one added harmonic (12‐h periodicity) + Days after Julian day	Nest ID
M_2.10_	Fundamental period with two added harmonics (8‐h periodicity) + Days after Julian day	Nest ID
M_2.11_	Nest age + Ambient temperature	Nest ID
M_2.12_	Nest age	Nest ID
M_2.13_	Ambient temperature	Nest ID
M_2.14_	Days after Julian day	Nest ID
M_2.15_	Days after Julian Day + Ambient temperature	Nest ID
*Response: Extended neck posture (logit link)*		
M_3.1_	(Null model)	Nest ID
M_3.2_	Fundamental period (24‐h)	Nest ID
M_3.3_	Fundamental period with one added harmonic (12‐h periodicity)	Nest ID
M_3.4_	Fundamental period with two added harmonics (8‐h periodicity)	Nest ID
M_3.5_	Fundamental period (24‐h) + Nest age	Nest ID
M_3.6_	Fundamental period with one added harmonic (12‐h periodicity) + Nest age	Nest ID
M_3.7_	Fundamental period with two added harmonics (8‐h periodicity) + Nest age	Nest ID
M_3.8_	Fundamental period (24‐h) + Days after Julian day	Nest ID
M_3.9_	Fundamental period with one added harmonic (12‐h periodicity) + Days after Julian day	Nest ID
M_3.10_	Fundamental period with two added harmonics (8‐h periodicity) + Days after Julian day	Nest ID
M_3.11_	Nest age + Ambient temperature	Nest ID
M_3.12_	Nest age	Nest ID
M_3.13_	Ambient temperature	Nest ID
M_3.14_	Days after Julian day	Nest ID
M_3.15_	Days after Julian day + Ambient temperature	Nest ID
*Response: log*_*10*_ *(Incubation bout length) (identity link)*		
M_4.1_	(Null model)	Nest ID
M_4.2_	Fundamental period (24‐h)	Nest ID
M_4.3_	Fundamental period with one added harmonic (12‐h periodicity)	Nest ID
M_4.4_	Fundamental period with two added harmonics (8‐h periodicity)	Nest ID
M_4.5_	Fundamental period (24‐h) + Nest age	Nest ID
M_4.6_	Fundamental period with one added harmonic (12‐h periodicity) + Nest age	Nest ID
M_4.7_	Fundamental period with two added harmonics (8‐h periodicity) + Nest age	Nest ID
M_4.8_	Fundamental period (24‐h) + Days after Julian day	Nest ID
M_4.9_	Fundamental period with one added harmonic (12‐h periodicity) + Days after Julian day	Nest ID
M_4.10_	Fundamental period with two added harmonics (8‐h periodicity) + Days after Julian day	Nest ID
M_4.11_	Fundamental period (24‐h) + MaleFemale	Nest ID
M_4.12_	Fundamental period with one added harmonic (12‐h periodicity) + MaleFemale	Nest ID
M_4.13_	Fundamental period with two added harmonics (8‐h periodicity) + MaleFemale	Nest ID
M_4.14_	Fundamental period (24‐h) + Nest age + MaleFemale	Nest ID
M_4.15_	Fundamental period with one added harmonic (12‐h periodicity) + Nest age + MaleFemale	Nest ID
M_4.16_	Fundamental period with two added harmonics (8‐h periodicity) + Nest age + MaleFemale	Nest ID
M_4.17_	Fundamental period (24‐h) + Days after Julian Day + MaleFemale	Nest ID
M_4.18_	Fundamental period with one added harmonic (12‐h periodicity) + Days after Julian day + MaleFemale	Nest ID
M_4.19_	Fundamental period with two added harmonics (8‐h periodicity) + Days after Julian day + MaleFemale	Nest ID
M_4.20_	Nest age + Ambient temperature	Nest ID
M_4.21_	Nest age	Nest ID
M_4.22_	Ambient temperature	Nest ID
M_4.23_	Nest age + Ambient temperature + MaleFemale	Nest ID
M_4.24_	Nest age + MaleFemale	Nest ID
M_4.25_	MaleFemale	Nest ID
M_4.26_	Ambient temperature + MaleFemale	Nest ID
M_4.27_	Days after Julian day + Ambient temperature	Nest ID
M_4.28_	Days after Julian day	Nest ID
M_4.29_	Days after Julian day + Ambient temperature + MaleFemale	Nest ID
M_4.30_	Days after Julian day + MaleFemale	Nest ID
**Breeding Attempt A3**		
*Response: Presence of incubating bird (logit link)*		
M_1.1_	(Null model)	Nest ID
M_1.2_	Fundamental period (24‐h)	Nest ID
M_1.3_	Fundamental period with one added harmonic (12‐h periodicity)	Nest ID
M_1.4_	Fundamental period with two added harmonics (8‐h periodicity)	Nest ID
M_1.5_	Fundamental period (24‐h) + Nest age	Nest ID
M_1.6_	Fundamental period with one added harmonic (12‐h periodicity) + Nest age	Nest ID
M_1.7_	Fundamental period with two added harmonics (8‐h periodicity) + Nest age	Nest ID
M_1.8_	Nest age + Ambient temperature	Nest ID
M_1.9_	Nest age	Nest ID
M_1.10_	Ambient temperature	Nest ID
*Response: Presence of male incubating (logit link)*		
M_2.1_	(Null model)	Nest ID
M_2.2_	Fundamental period (24‐h)	Nest ID
M_2.3_	Fundamental period with one added harmonic (12‐h periodicity)	Nest ID
M_2.4_	Fundamental period with two added harmonics (8‐h periodicity)	Nest ID
M_2.5_	Fundamental period (24‐h) + Nest age	Nest ID
M_2.6_	Fundamental period with one added harmonic (12‐h periodicity) + Nest age	Nest ID
M_2.7_	Fundamental period with two added harmonics (8‐h periodicity) + Nest age	Nest ID
M_2.8_	Nest age + Ambient temperature	Nest ID
M_2.9_	Nest age	Nest ID
M_2.10_	Ambient temperature	Nest ID
*Response: Extended neck posture (logit link)*		
M_3.1_	(Null model)	Nest ID
M_3.2_	Fundamental period (24‐h)	Nest ID
M_3.3_	Fundamental period with one added harmonic (12‐h periodicity)	Nest ID
M_3.4_	Fundamental period with two added harmonics (8‐h periodicity)	Nest ID
M_3.5_	Fundamental period (24‐h) + Nest age	Nest ID
M_3.6_	Fundamental period with one added harmonic (12‐h periodicity) + Nest age	Nest ID
M_3.7_	Fundamental period with two added harmonics (8‐h periodicity) + Nest age	Nest ID
M_3.8_	Nest age + Ambient temperature	Nest ID
M_3.9_	Nest age	Nest ID
M_3.10_	Ambient temperature	Nest ID
*Response: log*_*10*_ *(Incubation bout length) (identity link)*		
M_4.1_	(Null model)	Nest ID
M_4.2_	Fundamental period (24‐h)	Nest ID
M_4.3_	Fundamental period with one added harmonic (12‐h periodicity)	Nest ID
M_4.4_	Fundamental period with two added harmonics (8‐h periodicity)	Nest ID
M_4.5_	Fundamental period (24‐h) + Nest age	Nest ID
M_4.6_	Fundamental period with one added harmonic (12‐h periodicity) + Nest age	Nest ID
M_4.7_	Fundamental period with two added harmonics (8‐h periodicity) + Nest age	Nest ID
M_4.8_	Fundamental period (24‐h) + MaleFemale	Nest ID
M_4.9_	Fundamental period with one added harmonic (12‐h periodicity) + MaleFemale	Nest ID
M_4.10_	Fundamental period with two added harmonics (8‐h periodicity) + MaleFemale	Nest ID
M_4.11_	Fundamental period (24‐h) + Nest age + MaleFemale	Nest ID
M_4.12_	Fundamental period with one added harmonic (12‐h periodicity) + Nest Age + MaleFemale	Nest ID
M_4.13_	Fundamental period with two added harmonics (8‐h periodicity) + Nest Age + MaleFemale	Nest ID
M_4.14_	Nest age + Ambient temperature	Nest ID
M_4.15_	Nest age	Nest ID
M_4.16_	Ambient temperature	Nest ID
M_4.17_	Nest age + Ambient temperature + MaleFemale	Nest ID
M_4.18_	Nest age + MaleFemale	Nest ID
M_4.19_	MaleFemale	Nest ID
M_4.20_	Ambient temperature + MaleFemale	Nest ID

**TABLE A3:** Generalised linear mixed models for different dependent variables of black skimmer incubation behaviour as classified from 1‐min time lapse images, compared and ranked by AIC_c_.

Model	*K*	AICc	ΔAICc	*W*	Cumulative *W*	LL
Response: Presence of incubating bird (logit link)						
**Breeding Attempt A2**						
M_1.7_	9	11,347.67	0.00	0.91	0.91	−5664.83
M_1.10_	9	11,352.25	4.58	0.09	1.00	−5667.12
M_1.4_	8	11,361.51	13.84	0.00	1.00	−5672.75
M_1.6_	7	11,406.49	58.82	0.00	1.00	−5696.24
M_1.9_	7	11,411.11	63.44	0.00	1.00	−5698.55
M_1.3_	6	11,420.20	72.53	0.00	1.00	−5704.10
M_1.5_	5	11,665.45	317.78	0.00	1.00	−5827.72
M_1.8_	5	11,670.00	322.33	0.00	1.00	−5830.00
M_1.2_	4	11,679.01	331.35	0.00	1.00	−5835.51
M_1.11_	4	11,722.72	375.05	0.00	1.00	−5857.36
M_1.15_	4	11,725.76	378.09	0.00	1.00	−5858.88
M_1.13_	3	11,728.35	380.68	0.00	1.00	−5861.18
M_1.12_	3	11,735.61	387.94	0.00	1.00	−5864.80
M_1.14_	3	11,739.88	392.22	0.00	1.00	−5866.94
M_1.1_	2	11,747.51	399.84	0.00	1.00	−5871.75
**Breeding Attempt A3**						
M_1.7_	9	12,884.47	0.00	1.00	1.00	−6433.23
M_1.6_	7	12,905.97	21.51	0.00	1.00	−6445.98
M_1.5_	5	13,040.39	155.93	0.00	1.00	−6515.19
M_1.4_	8	13,101.94	217.47	0.00	1.00	−6542.97
M_1.3_	6	13,123.37	238.91	0.00	1.00	−6555.69
M_1.8_	4	13,164.18	279.71	0.00	1.00	−6578.09
M_1.9_	3	13,252.18	367.71	0.00	1.00	−6623.09
M_1.2_	4	13,256.94	372.48	0.00	1.00	−6624.47
M_1.10_	3	13,360.10	475.63	0.00	1.00	−6677.05
M_1.1_	2	13,480.33	595.86	0.00	1.00	−6738.16
Response: Presence of male incubating (logit link)						
**Breeding Attempt A2**						
M_2.7_	9	40,462.11	0.00	0.95	0.95	−20,222.05
M_2.10_	9	40,468.01	5.89	0.05	1.00	−20,225.00
M_2.4_	8	40,563.30	101.19	0.00	1.00	−20,273.65
M_2.6_	7	40,611.45	149.33	0.00	1.00	−20,298.72
M_2.9_	7	40,616.50	154.38	0.00	1.00	−20,301.25
M_2.5_	5	40,644.74	182.63	0.00	1.00	−20,317.37
M_2.11_	4	40,646.49	184.37	0.00	1.00	−20,319.24
M_2.8_	5	40,649.99	187.88	0.00	1.00	−20,320.00
M_2.15_	4	40,651.84	189.72	0.00	1.00	−20,321.92
M_2.12_	3	40,668.16	206.05	0.00	1.00	−20,331.08
M_2.14_	3	40,673.36	211.25	0.00	1.00	−20,333.68
M_2.3_	6	40,714.38	252.27	0.00	1.00	−20,351.19
M_2.13_	3	40,722.86	260.75	0.00	1.00	−20,358.43
M_2.2_	4	40,751.63	289.51	0.00	1.00	−20,371.81
M_2.1_	2	40,780.44	318.33	0.00	1.00	−20,388.22
**Breeding Attempt A3**						
M_2.7_	9	37,933.89	0.00	1.00	1.00	−18,957.94
M_2.6_	7	37,997.37	63.47	0.00	1.00	−18,991.68
M_2.5_	5	38,001.57	67.68	0.00	1.00	−18,995.79
M_2.8_	4	38,039.58	105.69	0.00	1.00	−19,015.79
M_2.9_	3	38,048.49	114.60	0.00	1.00	−19,021.24
M_2.4_	8	38,118.85	184.96	0.00	1.00	−19,051.42
M_2.3_	6	38,181.92	248.02	0.00	1.00	−19,084.96
M_2.2_	4	38,189.60	255.70	0.00	1.00	−19,090.80
M_2.10_	3	38,224.64	290.75	0.00	1.00	−19,109.32
M_2.1_	2	38,227.72	293.83	0.00	1.00	−19,111.86
Response: Extended neck posture (logit link)						
**Breeding Attempt A2**						
M_3.10_	9	21,149.38	0.00	0.93	0.93	−10,565.69
M_3.7_	9	21,154.58	5.20	0.07	1.00	−10,568.29
M_3.11_	4	21,198.17	48.80	0.00	1.00	−10,595.09
M_3.15_	4	21,199.49	50.12	0.00	1.00	−10,595.75
M_3.13_	3	21,235.06	85.68	0.00	1.00	−10,614.53
M_3.9_	7	21,304.32	154.95	0.00	1.00	−10,645.16
M_3.6_	7	21,309.45	160.08	0.00	1.00	−10,647.73
M_3.4_	8	21,621.48	472.10	0.00	1.00	−10,802.74
M_3.8_	5	21,773.03	623.65	0.00	1.00	−10,881.52
M_3.3_	6	21,773.38	624.00	0.00	1.00	−10,880.69
M_3.5_	5	21,777.91	628.54	0.00	1.00	−10,883.96
M_3.2_	4	22,232.37	1082.99	0.00	1.00	−11,112.18
M_3.14_	3	23,964.96	2815.59	0.00	1.00	−11,979.48
M_3.12_	3	23,970.91	2821.53	0.00	1.00	−11,982.45
M_3.1_	2	24,371.73	3222.35	0.00	1.00	−12,183.86
**Breeding Attempt A3**						
M_3.7_	9	20,272.42	0.00	0.61	0.61	−10,127.21
M_3.4_	8	20,273.34	0.92	0.39	1.00	−10,128.67
M_3.6_	7	20,296.28	23.86	0.00	1.00	−10,141.14
M_3.3_	6	20,297.38	24.96	0.00	1.00	−10,142.69
M_3.8_	4	20,510.45	238.03	0.00	1.00	−10,251.23
M_3.10_	3	20,512.16	239.74	0.00	1.00	−10,253.08
M_3.2_	4	20,712.93	440.51	0.00	1.00	−10,352.46
M_3.5_	5	20,714.12	441.70	0.00	1.00	−10,352.06
M_3.9_	3	25,901.16	5628.74	0.00	1.00	−12,947.58
M_3.1_	2	25,903.66	5631.24	0.00	1.00	−12,949.83
Response: log_10_(Incubation bout length) (identity link)						
**Breeding Attempt A2**						
M_4.19_	11	1628.80	0.00	0.94	0.94	−803.31
M_4.16_	11	1634.68	5.88	0.05	0.99	−806.25
M_4.13_	10	1637.60	8.80	0.01	1.00	−808.73
M_4.10_	10	1644.02	15.21	0.00	1.00	−811.93
M_4.17_	7	1645.68	16.88	0.00	1.00	−815.80
M_4.18_	9	1647.97	19.16	0.00	1.00	−814.92
M_4.7_	10	1650.08	21.28	0.00	1.00	−814.96
M_4.14_	7	1651.28	22.48	0.00	1.00	−818.60
M_4.4_	9	1653.18	24.38	0.00	1.00	−817.53
M_4.11_	6	1653.30	24.49	0.00	1.00	−820.62
M_4.15_	9	1653.58	24.77	0.00	1.00	−817.73
M_4.12_	8	1655.59	26.79	0.00	1.00	−819.74
M_4.29_	6	1657.76	28.96	0.00	1.00	−822.85
M_4.26_	5	1658.65	29.85	0.00	1.00	−824.30
M_4.8_	6	1659.68	30.88	0.00	1.00	−823.81
M_4.23_	6	1660.37	31.56	0.00	1.00	−824.16
M_4.9_	8	1662.13	33.33	0.00	1.00	−823.02
M_4.5_	6	1665.46	36.66	0.00	1.00	−826.70
M_4.2_	5	1667.63	38.82	0.00	1.00	−828.79
M_4.6_	8	1667.92	39.11	0.00	1.00	−825.91
M_4.3_	7	1670.08	41.28	0.00	1.00	−828.00
M_4.27_	5	1670.80	41.99	0.00	1.00	−830.38
M_4.22_	4	1671.78	42.98	0.00	1.00	−831.88
M_4.20_	5	1673.49	44.68	0.00	1.00	−831.72
M_4.30_	5	1700.52	71.72	0.00	1.00	−845.24
M_4.24_	5	1706.60	77.80	0.00	1.00	−848.28
M_4.25_	4	1708.84	80.03	0.00	1.00	−850.40
M_4.28_	4	1713.96	85.15	0.00	1.00	−852.97
M_4.21_	4	1720.21	91.41	0.00	1.00	−856.09
M_4.1_	3	1722.52	93.72	0.00	1.00	−858.25
**Breeding Attempt A3**						
M_4.13_	11	1960.55	0.00	0.78	0.78	−969.19
M_4.7_	10	1963.33	2.78	0.19	0.97	−971.60
M_4.11_	7	1968.37	7.82	0.02	0.99	−977.15
M_4.5_	6	1970.79	10.25	0.00	0.99	−979.37
M_4.17_	6	1971.80	11.25	0.00	1.00	−979.87
M_4.12_	9	1972.10	11.56	0.00	1.00	−976.99
M_4.14_	5	1974.11	13.56	0.00	1.00	−982.03
M_4.6_	8	1974.54	13.99	0.00	1.00	−979.22
M_4.10_	10	1993.70	33.16	0.00	1.00	−986.78
M_4.4_	9	1995.60	35.05	0.00	1.00	−988.74
M_4.8_	6	2001.50	40.95	0.00	1.00	−994.72
M_4.20_	5	2001.67	41.13	0.00	1.00	−995.82
M_4.2_	5	2003.07	42.53	0.00	1.00	−996.52
M_4.16_	4	2003.28	42.73	0.00	1.00	−997.63
M_4.9_	8	2004.94	44.39	0.00	1.00	−994.42
M_4.3_	7	2006.53	45.98	0.00	1.00	−996.23
M_4.18_	5	2138.99	178.45	0.00	1.00	−1064.48
M_4.15_	4	2139.57	179.02	0.00	1.00	−1065.77
M_4.1_	3	2179.29	218.75	0.00	1.00	−1086.64
M_4.19_	4	2179.44	218.89	0.00	1.00	−1085.71

*Note*: Two distinct breeding attempts were analysed separately (A2 and A3). Each model included Nest ID as a random effect, the null model always being denoted by M_.1_. The predictor variables and parameter estimates for the best ranking models from each set of models are presented in Table 1, with the same model numbers for reference. Models with ambient temperature as the predictor variable are presented in Table A4, also with model numbers for reference.

**TABLE A4:** Generalised linear mixed model parameter estimates with ambient temperature as the explanatory variable for black skimmer incubation behaviour as classified from 1‐min time lapse images.

	A2					A3			
	Estimate	SE	Lwr 95% CI	Upr 95% CI		Estimate	SE	Lwr 95% CI	Upr 95% CI
*Response: Presence of incubating bird (logit link)*									
M_1.13_					M_1.10_				
(Intercept)	2.4236	0.2833	1.8684	2.9789	(Intercept)	4.7510	0.2075	4.3444	5.1577
Ambient temperature	0.0357	0.0079	0.0203	0.0512	Ambient temperature	−0.0668	0.0060	−0.0784	−0.0551
*Response: Presence of male incubating (logit link)*									
M_2.13_					M_2.10_				
(Intercept)	−0.8973	0.1006	−1.0944	−0.7002	(Intercept)	−0.5045	0.1281	−0.7557	−0.2533
Ambient temperature	0.0241	0.0031	0.0180	0.0302	Ambient temperature	0.0069	0.0030	0.0009	0.0129
*Response: Extended neck posture (logit link)*									
M_3.13_					M_3.10_				
(Intercept)	−8.8323	0.1954	−9.2152	−8.4493	(Intercept)	−10.1354	0.1914	−10.5109	−9.7599
Ambient temperature	0.2588	0.0049	0.2492	0.2684	Ambient temperature	0.3062	0.0047	0.2969	0.3154
*Response: log*_*10*_ *(Incubation bout length) (identity link)*									
M_4.22_					M_4.16_				
(Intercept)	1.6764	0.0835	1.5127	1.8401	(Intercept)	2.0427	0.0813	1.8834	2.2019
Ambient temperature	−0.0216	0.0029	−0.0274	−0.0158	Ambient temperature	−0.0371	0.0027	−0.0424	−0.0318

*Note*: None of these models were within ≤Δ2.0 AIC_c_ but are presented separately to demonstrate the effect of temperature in addition to that of the diel cycle. Cosinor functions testing the effect of time of day were not included in the same models as temperature due to high collinearity. Two distinct breeding attempts were analysed out of a total of three which occurred during study season, the second (A2) is presented on the left and the third (A3) on the right. Each model included Nest ID as a random effect. Model numbers are included so reference can be made to AIC_c_ ranking tables in Table A3.

**TABLE A5:** Results from two‐sided and one‐sided Mann–Whitney *U*‐tests for paired data with Bonferroni correction on temperatures recorded inside black skimmer nests and on the beach surface using *i*Buttons®.

Hour	Two‐sided		One‐sided: Greater		One‐sided: Less	
	V statistic	*p*‐Value	V statistic	*p*‐Value	V statistic	*p*‐Value
**Breeding Attempt A2**						
0	595.00	.00	595.00	.00	595.00	1.00
1	595.00	.00	595.00	.00	595.00	1.00
2	561.00	.00	561.00	.00	561.00	1.00
3	561.00	.00	561.00	.00	561.00	1.00
4	561.00	.00	561.00	.00	561.00	1.00
5	561.00	.00	561.00	.00	561.00	1.00
6	561.00	.00	561.00	.00	561.00	1.00
7	528.00	.00	528.00	.00	528.00	1.00
8	220.00	.42	220.00	.80	220.00	.21
9	24.00	.00	24.00	1.00	24.00	.00
10	7.00	.00	7.00	1.00	7.00	.00
11	3.00	.00	3.00	1.00	3.00	.00
12	5.00	.00	5.00	1.00	5.00	.00
13	4.00	.00	4.00	1.00	4.00	.00
14	6.00	.00	6.00	1.00	6.00	.00
15	21.00	.00	21.00	1.00	21.00	.00
16	182.50	.08	182.50	.96	182.50	.04
17	525.00	.00	525.00	.00	525.00	1.00
18	595.00	.00	595.00	.00	595.00	1.00
19	595.00	.00	595.00	.00	595.00	1.00
20	595.00	.00	595.00	.00	595.00	1.00
21	595.00	.00	595.00	.00	595.00	1.00
22	595.00	.00	595.00	.00	595.00	1.00
23	595.00	.00	595.00	.00	595.00	1.00
**Breeding Attempt A3**						
0	1326.00	.00	1326.00	.00	1326.00	1.00
1	1326.00	.00	1326.00	.00	1326.00	1.00
2	1275.00	.00	1275.00	.00	1275.00	1.00
3	1275.00	.00	1275.00	.00	1275.00	1.00
4	1275.00	.00	1275.00	.00	1275.00	1.00
5	1275.00	.00	1275.00	.00	1275.00	1.00
6	1275.00	.00	1275.00	.00	1275.00	1.00
7	1258.50	.00	1258.50	.00	1258.50	1.00
8	179.00	.00	179.00	1.00	179.00	.00
9	53.00	.00	53.00	1.00	53.00	.00
10	10.00	.00	10.00	1.00	10.00	.00
11	10.00	.00	10.00	1.00	10.00	.00
12	9.00	.00	9.00	1.00	9.00	.00
13	4.00	.00	4.00	1.00	4.00	.00
14	0.00	.00	0.00	1.00	0.00	.00
15	0.00	.00	0.00	1.00	0.00	.00
16	4.00	.00	4.00	1.00	4.00	.00
17	801.50	.06	801.50	.03	801.50	.97
18	1326.00	.00	1326.00	.00	1326.00	1.00
19	1326.00	.00	1326.00	.00	1326.00	1.00
20	1326.00	.00	1326.00	.00	1326.00	1.00
21	1326.00	.00	1326.00	.00	1326.00	1.00
22	1326.00	.00	1326.00	.00	1326.00	1.00
23	1326.00	.00	1326.00	.00	1326.00	1.00

*Note*: Tests were carried out per hour and for two breeding attempts (A2: 3 nests and 802 h; A3: 5 nests and 1209 h). One‐sided tests were carried out testing whether nest temperature was greater or less than the beach surface respectively.
